# De-intensification of postoperative radiotherapy in head and neck cancer irrespective of human papillomavirus status—results of a prospective multicenter phase II trial (DIREKHT Trial)

**DOI:** 10.3389/fonc.2024.1447123

**Published:** 2024-08-19

**Authors:** Marlen Haderlein, Jens von der Grün, Panagiotis Balermpas, Claus Rödel, Matthias G. Hautmann, Felix Steger, Christopher Bohr, Thomas Hehr, Carmen Stromberger, Volker Budach, Markus Schymalla, Rita Engenhart-Cabillic, Lukas Kocik, Hans Geinitz, Ursula Nestle, Gunter Klautke, Claudia Scherl, Christine Gall, Benjamin Frey, Philipp Schubert, Sabine Semrau, Oliver Ott, Marco Kesting, Heinrich Iro, Sarina K. Mueller, Rainer Fietkau

**Affiliations:** ^1^ Department of Radiotherapy and Radiation Oncology, University Hospital Erlangen, Friedrich-Alexander-Universität Erlangen-Nürnberg, Erlangen, Germany; ^2^ Comprehensive Cancer Center Erlangen-EMN (CCC ER-EMN), Universitätsklinikum Erlangen, Friedrich-Alexander-Universität Erlangen-Nürnberg, Erlangen, Germany; ^3^ Department of Radiotherapy and Radiation Oncology, University Hospital Frankfurt, Goethe-Universitat Frankfurt am Main, Frankfurt am Main, Germany; ^4^ Department of Radiation Oncology, Zurich University Hospital, Zurich, Switzerland; ^5^ Department of Radiotherapy and Radiation Oncology, University Hospital of Regensburg, Regensburg, Germany; ^6^ Department of Otorhinolaryngology, University Hospital Regensburg, Regensburg, Germany; ^7^ Department of Radiotherapy and Radiation Oncology, Marienhospital, Stuttgart, Germany; ^8^ Department of Radiotherapy and Radiation Oncology, Charité - Universitätsmedizin Berlin, corporate member of Freie Universität Berlin and Humboldt-Universität zu Berlin, Berlin, Germany; ^9^ Department of Radiotherapy and Radiation Oncology, University Hospital of Marburg, Marburg, Germany; ^10^ Department of Radiation Oncology and Radiotherapy, Ordensklinikum Linz Barmherzige Schwestern, Linz, Austria; ^11^ Department of Radiotherapy and Radiation Oncology, Kliniken Maria Hilf, Moenchengladbach, Germany; ^12^ Department of Radiation Oncology, Freiburg University Medical Center, Freiburg, Germany; ^13^ Department of Radiation Oncology, Chemnitz Hospital, Chemnitz, Germany; ^14^ Department of Otorhinolaryngology, Head and Neck Surgery, University Hospital Mannheim, Medical Faculty Mannheim, Heidelberg University, Mannheim, Germany; ^15^ Department of Medical Informatics, Biometry and Epidemiology, Friedrich-Alexander-Universität Erlangen-Nürnberg, Erlangen, Germany; ^16^ Department of Oral and Maxillofacial Surgery, University Hospital Erlangen, Erlangen, Germany; ^17^ Department of Otolaryngology - Head & Neck Surgery, University Hospital Erlangen, Friedrich-Alexander-Universität Erlangen-Nürnberg, Erlangen, Germany

**Keywords:** head and neck cancer, radiotherapy, HPV, de-intensification, postoperative

## Abstract

**Background:**

Current standard treatment concepts in head and neck squamous cell carcinoma (HNSCC) are based on former studies using 2D and 3D treatment plans. However, modern radiation techniques allow for a more precise and individual dose application. Therefore, in a clearly defined patient population, de-intensified risk-adapted radiation is investigated.

**Methods:**

Patients with newly diagnosed HNSCC after surgery (with resection margins ≥1 mm and cM0) with the following tumor stages (TNM 7th Edition) were eligible for the study: oral cavity, oropharynx, or larynx: pT1–3, pN0–pN2b; hypopharynx: pT1–2, pN1. The patients should have either a low risk of local recurrence [≤pT2, resection margin ≥5 mm, no peritumoral lymphangiosis (L0), and no perineural invasion] or contralateral lymph node metastasis (≤3 ipsilateral lymph node metastases, in case of well-lateralized oropharyngeal or oral cavity cancer contralateral cN0, otherwise pN0). Patients were assigned to three different treatment regimes with reduction of the treated volume, radiation dose, or both, according to tumor stage and results of surgery performed. The primary objective was to show an LRR of <10% after 2 years.

**Findings:**

A total of 150 patients were enrolled. Tumor localizations were as follows: *n* = 53 (35.3%), oral cavity; *n* = 94 (62.7%), oropharynx (82% HPV-positive); *n* = 2 (1.3%), hypopharynx; and *n* = 1 (0.7%), larynx. A total of 61 patients (41.0%) were stage IVA, 81 (54.0%) were stage III, and 8 (5.3%) were stage II. Median follow-up was 36 months. Cumulative incidence of 2y-LRR was 5.6% (95% CI: 1.7%–9.2%) in the whole study population and 14.1% (95% CI: 3.8%–23.2%) in patients with oral cavity cancer. Cumulative incidence of 2y-LRR in non-irradiated or dose-reduced regions was 3.5% (95% CI: 0.4%–6.5%). After 2 years, disease-free survival was 92% (95% CI: 87%–96%) and overall survival was 94% (95% CI: 90%–98%) for the complete study cohort. Acute III° toxicity was as follows: dysphagia, 30%; xerostomia, 7%; mucositis, 19%; and dermatitis, 4%. Dysphagia and xerostomia decrease over time. After 27 months, late dysphagia III° and xerostomia II° were 1% and 9%, respectively.

**Interpretation:**

The study met its primary objective. De-intensification of postoperative radiotherapy irrespective of HPV status in a predefined patient population is associated with a favorable toxicity profile without compromising LRR. In an unplanned subgroup analysis, a significantly increased risk of LRR was observed in patients with oral cavity cancer. In these patients, de-intensified radiotherapy should be applied with caution.

## Background

In low- and intermediate-risk patient populations with head and neck cancer, 5-year locoregional control rates of over 90% have been achieved, but approximately 30% of the patients suffer from grade III therapy-related side effects, like xerostomia, dysphagia, and trismus, leading to a reduced quality of life (1–7).

Long-term toxicity is highly relevant for cancer survivors. In the last decades, head and neck surgeons significantly de-intensified the extent of surgery in an effort to preserve functionality, e.g., preferring selective or modified radical neck dissection over radical neck dissections. Modern radiation techniques allow a more precise and individual dose application. However, current standard radiotherapy treatment concepts in head and neck squamous cell carcinoma (HNSCC) are based on former studies using 2D and 3D treatment plans. To achieve reduced long-term toxicity rates while maintaining locoregional control, individualized risk-adapted therapy de-intensification is of great interest.

To date, several studies investigated de-intensification of therapy in HPV-associated head and neck carcinomas, but only few studies included patients with HPV-negative head and neck cancer. There are only small prospective studies (8–10) and some retrospective studies (11–13) investigating the possibility of treating ipsilateral elective neck nodes only in the postoperative situation of head and neck cancer. Moreover, the primary tumor region was usually treated with a dose of up to 60–66 Gy (14–16).

To overcome this shortcoming, the objective of this study, the prospective non-randomized multicentric DIREKHT-Trial, was the investigation of an individual de-escalation of the radiotherapy dose and volume in a predefined patient population irrespective of HPV status. The aim was to reduce late toxicity without compromising the oncological outcome defined as locoregional control.

## Methods

We performed a prospective multicenter non-randomized phase II trial in a highly selected patient population. Patients should have either a low risk for local recurrence or a low risk for contralateral neck metastases as defined below.

Eligibility criteria were as follows:

- Newly diagnosed HNSCC after resection of the primary tumor and neck dissection.
- No distant metastases.
- Resection margin (R) ≥1 mm.
- According to TNM 7th Edition, the following tumor stages were eligible: oral cavity, oropharynx, or larynx: pT1–3, pN0–pN2b; hypopharynx: pT1–2, pN1.
- Moreover, patients had to fulfill one or both of the following criteria:

► Either low risk for local recurrence defined as ≤pT2 and resection margin ≥ 5 mm and no peritumoral lymphangiosis (L0) and PNI negative (without perineural invasion).

or

► Low risk for contralateral neck node metastases defined as ≤3 ipsilateral lymph node metastases in the setting of adequate contralateral neck dissection of at least six negative nodes (according to WHO) or well-lateralized oropharynx or oral cavity cancers with a minimum of 5 mm from midline in which contralateral neck dissection was not performed.

HPV status in patients with oropharyngeal cancer was tested, but study inclusion and treatment de-intensification were independent of HPV status.

Depending on individual risk factors (low risk for local and/or contralateral neck recurrence), three different de-intensification regimes were possible:

A. Dose reduction in primary tumor region to 56 Gy

B. Omitting elective contralateral neck

C. Dose reduction in primary tumor region and omitting contralateral elective neck

**Figure 1:**
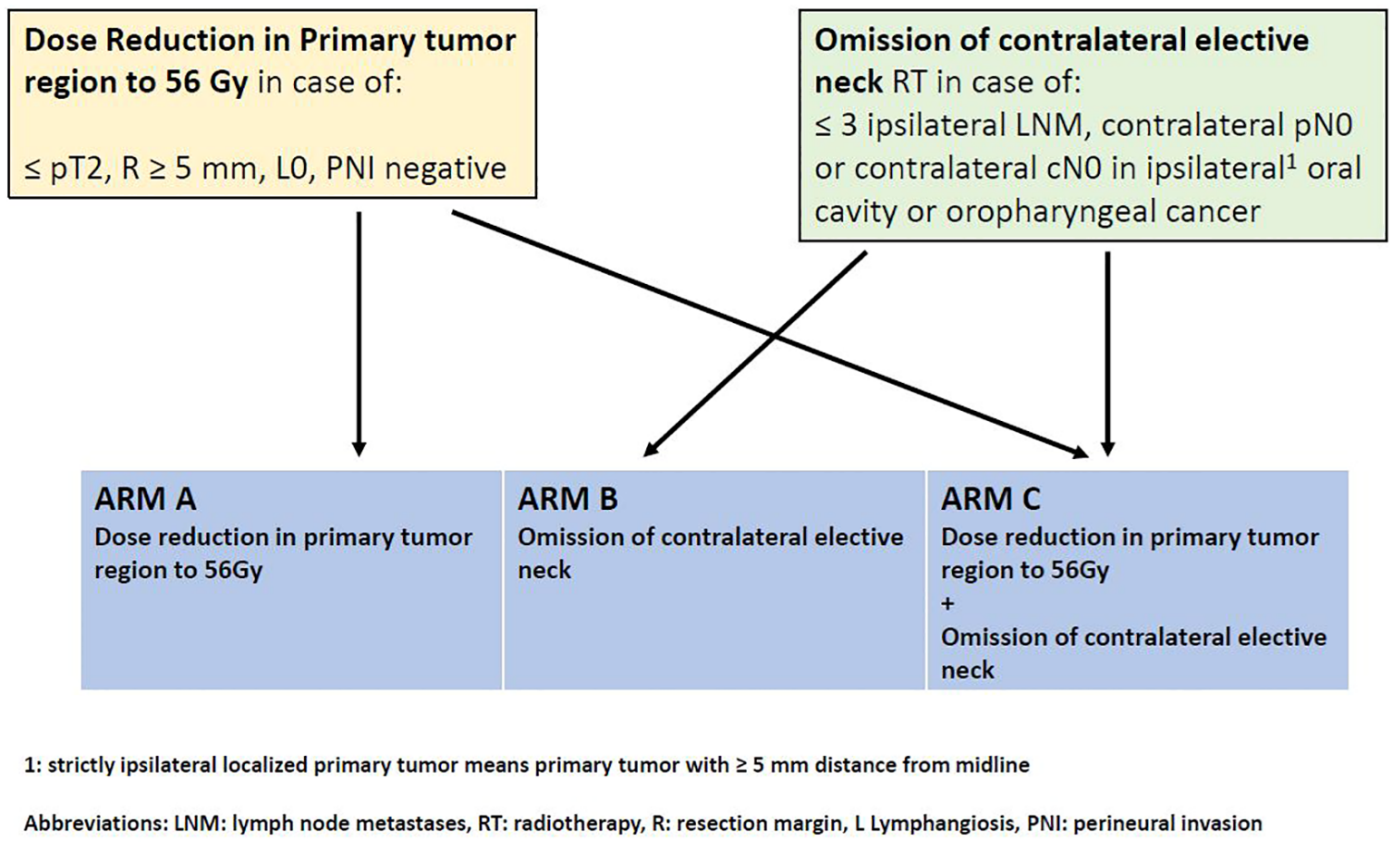
Study scheme.

For more detailed information, see **Figure 1**.

The study protocol was approved by the local ethics committee No.195_14B and registered in clinicaltrials.gov NCT02528955.

### Target volume definition, dose prescription, and radiotherapy planning

Target volume definition and dose prescription were already published in detail (17).

In short, target volumes and dose prescriptions were as follows:

Clinical target volume (CTV)1 included the former primary tumor region or space after resection (in case of ≥pT3 L1 Pn1 or resection margin <5 mm) and lymph node levels with resected lymph node metastases with extranodal extension (ENE).

CTV2 always included the former primary tumor region or space after resection and lymph node levels with resected lymph node metastases.

CTV3, in addition to CTV2, includes elective neck nodes.

Planning target volume (PTV) 1, 2, and 3 resulted by giving a safety margin of 3–5 mm (according to individual setup errors) around each of CTV1, 2, and 3.

The prescribed dose in PTV1, PTV2, and PTV3 was 64 Gy, 56 Gy, and 50 Gy, respectively. Single fraction dose was 2 Gy. One fraction per day and five fractions per week were delivered.

Radiotherapy was delivered as intensity-modulated radiotherapy (IMRT) or volumetric-intensity modulated arc therapy (VMAT) with daily image guidance, using the standard of the institution, e.g., cone beam computer tomography (CBCT) or ExacTRac.

### Concurrent chemotherapy

Chemotherapy consisted of 5-FU 800 mg/m² BSA d1–5, cisplatin 20 mg/m² BSA d1–5, qd29 as used in the ARO96-3 trial (16) and was applied according to standard indications in the ARO96-3 trial (close resection margins ≤5 mm, lymph node metastases with ENE, ≥3 lymph node metastases).

In cisplatin-ineligible patients, carboplatin AUC1 d1–5 and 5-FU 800 mg/m² BSA d1–5, qd29 were applied.

### Endpoints

The primary endpoint was the LRR after 2 years. Based on previous results of the ARO96-3 study, a 2-year LRR of 10% was expected. In areas with de-intensified radiation, an additional 6% of recurrences was assumed.

The sample size was calculated based on the predicted width of the confidence interval in a binomial distributed hit rate assuming an LRR of 10% (with a likelihood of 80%). As the estimation of the 2-year LRR was based on cumulative incidence rates and the longitudinal character of the data, the calculated sample size was then raised by 15%.

Initially, the half-width of the confidence intervall was planned to be maximally 4.8%, which leads to a sample size of 200. However, the study protocol includes the option of early stopping at a sample size of 150 patients in case of delayed recruitment. Then, the half-width rises to 5.6%.

Secondary endpoints were overall/disease-free survival (OS/DFS) and late toxicity according to common toxicity criteria for adverse events (CTC-AE) version 4.0.

### Statistical analysis

Data were collected and restored in an electronic case report form (eCRF) called Secutrial.

The statistical analysis was carried out using the R software (version 4.2.2) (25).

Event times were calculated from the time of first diagnosis (defined as the date of biopsy of the primary tumor). DFS was defined as the absence of locoregional or distant disease recurrence and death from any cause.

We addressed the primary endpoint LRR and the secondary endpoints DFS and OS, as well as the cumulative incidence of distant metastases (DM) and the occurrence of secondary cancer(SC) by the Kaplan–Meier method estimating cumulative incidences and survival. The log-rank test was used for comparison.

As most recurrences occurred in oral cavity cancer patients treated according to de-intensification regime B, an exploratory analysis to identify risk factors for LRR was carried out in this subgroup.

## Results

Between November 2014 and April 2021, 150 patients were enrolled in eight different study centers in Germany and Austria. Seven (4.7%) patients received dose reduction in primary tumor region only (regime A); in 95 (63.3%) patients, radiotherapy of the elective contralateral neck was omitted (regime B), and in 48 (32.0%) patients, dose in the primary tumor region was reduced and radiotherapy of the elective contralateral neck was omitted (regime C).

Median age was 59 years (range, 21–81 years). Tumor localizations were as follows: *n* = 53 patients (35.3%), oral cavity; *n* = 94 patients (62.7%), oropharynx (82% HPV-positive); *n* = 2 patients (1.3%), hypopharynx; and *n* = 1 (0.7%) patients, larynx. A total of 61 patients (40.7%) were stage IVA, 81 (54.0%) were stage III, and 8 (5.3%) were stage II according to AJCC, Seventh Edition (2010). For more detailed information on patient characteristics, see **Table 1**.

**Table 1:** Patient characteristics.

		Whole collective (*N* = 150)	Oral cavity, de-intensification schedule B (*N* = 41)	Oropharynx (*N* = 94: *N* = 67: tonsillar cancer, *N* = 11 base of tongue, and *N* = 16 soft palate)
		No. of patients (%)	No. of patients (%)	No. of patients (%)
**Sex**	Male	112 (74.7%)	30 (73.2%)	70 (74.5%)
	Female	38 (25.3%)	11 (26.8%)	24 (25.5%)
**Age at diagnosis, years**	Median, IQR	59 [54;65]	59 [54;68]	59 [55;64]
**AJCC classification (TNM 7th edition)**	II	8 (5.3%)	2 (4.9%)	2 (2.1%)
	III	69 (46%)	30 (73.2%)	31 (33%)
	IVa	73 (48.7%)	8 (19.5%)	61 (64.9%)
**pT classification**	T1	45 (30%)	4 (9.8%)	38 (40.4%)
	T2	74 (49.3%)	17 (41.5%)	47 (50%)
	T3	31 (20.7%)	20 (48.8%)	9 (9.6%)
**pN classification**	N0	21 (14%)	13 (31.7%)	3 (3.2%)
	N1	56 (37.3%)	20 (48.8%)	30 (31.9%)
	N2a	26 (17.3%)	0 (0%)	26 (27.7%)
	N2b	47 (31.3%)	8 (19.5%)	35 (37.2%)
**Extranodal extension (ENE)**	Yes	34 (22.7%)	11 (26.8%)	22 (23.4%)
	No	116 (77.3%)	30 (73.2%)	72 (76.6%)
**Lymphangiosis**	L0	131 (87.3%)	32 (78%)	85 (90.4%)
	L1	19 (12.7%)	9 (22%)	9 (9.6%)
**Hemangiosis**	V0	147 (98%)	41 (100%)	92 (97.9%)
	V1	3 (2%)	0 (0%)	2 (2.1%)
**Perineural invasion**	PNI	131 (87.3%)	31 (75.6%)	85 (90.4%)
	Pn1	19 (12.7%)	10 (24.4%)	9 (9.6%)
**Grading**	G1	7 (4.7%)	4 (9.8%)	3 (3.2%)
	G2	59 (39.3%)	25 (61%)	25 (26.6%)
	G3	83 (55.3%)	12 (29.3%)	66 (70.2%)
	Unknown	1 (0.7%)	0 (0%)	0 (0%)
**Midline infiltration of primary tumor**	Yes	31 (20.7%)	13 (31.7%)	15 (16%)
	No	119 (79.3%)	28 (68.3%)	79 (84%)
**Resection margin**	≥1 mm, <5 mm	58 (38.7%)	22 (53.7%)	34 (36.2%)
	≥5 mm	92 (61.3%)	19 (46.3%)	60 (63.8%)
**HPV in oropharyngeal cancer**	Positive			77 (81.9%)
	Negative			15 (16%)
	Not defined			2 (2.1%)
**Neck dissection**	Only ipsilateral	58 (38.7%)	10 (24.4%)	42 (44.7%)
	Bilateral	92 (61.3%)	31 (75.6%)	52 (55.3%)
**Concurrent chemotherapy, *n***	Yes	56 (37.3%)	14 (34.1%)	39 (41.5%)
	No	94 (62.7%)	27 (65.9%)	55 (58.5%)

### Locoregional recurrences

**Figure 2:**
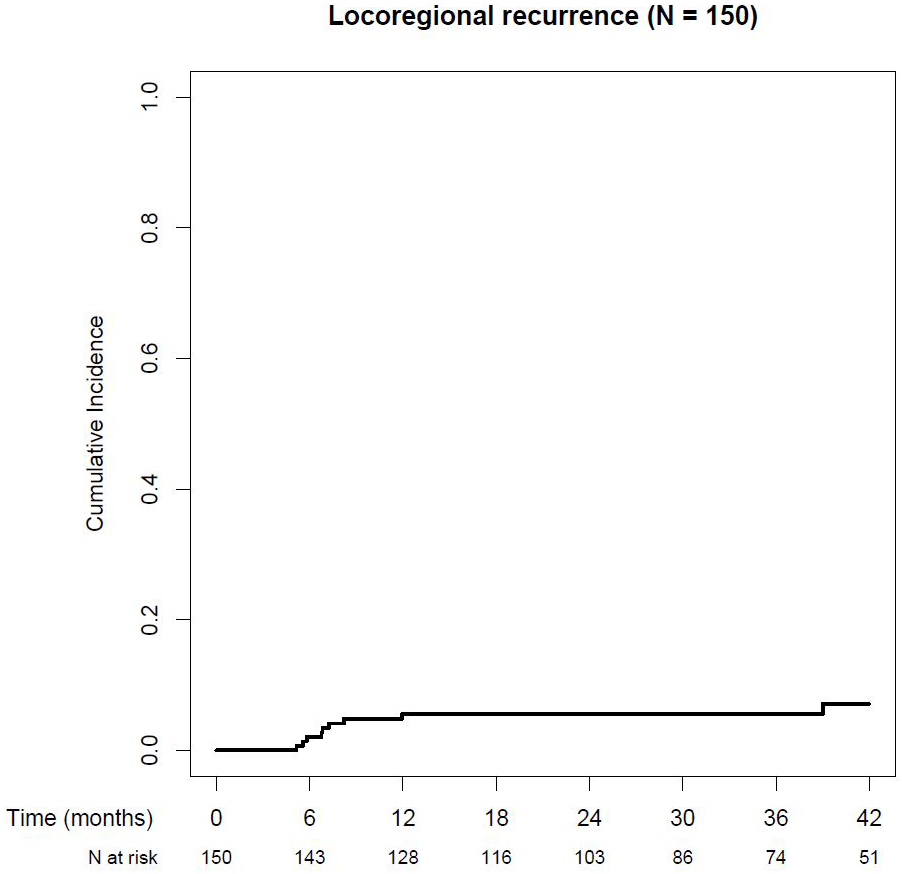
Cumulative locoregional recurrence rate.

Cumulative incidence of LRR after 1, 2, and 3 years was 5.6%, 5.6%, and 5.6% (95% CI: 1.7%–9.2%), respectively, in the whole study population (see **Figure 2**). For detailed information on locoregional recurrences, see **Table 2**.

**Table 2:** Detailed information on locoregional recurrences.

Therapy regime	Number of patients (*n*)	Number of LRR (*n*)	Localization of primary tumor	Localization of locoregional recurrences and additional information regarding midline infiltration of primary tumor and contralateral neck dissection (ND) (in red: recurrences in dose-reduced areas and non-irradiated regions)
A (Dose reduction in primary tumor region))	7	1	*N* = 1: oral cavity cancer	*N* = 1 (no midline infiltration, no contralateral ND): In-field local recurrence (in the dose-reduced primary tumor region that received 56 Gy)
B (Omission of contralateral elective neck nodes)	95	7	*N* = 7: oral cavity cancer	*N* = 1 (midline infiltration, contralateral ND): In-field local recurrence in the region that received 64 Gy plus regional recurrence in contralateral neck nodes (non-irradiated neck)
				*N* = 2 (no midline infiltration, no contralateral ND): Regional recurrence in contralateral neck node (non-irradiated neck)
				*N* = 3 (*N* = 1 midline infiltration, *N* = 2 contralateral ND): In-field local recurrence in the region that received 64 Gy
				*N* = 1 (no midline infiltration, no contralateral ND): In-field local recurrence in the region that received 64 Gy and ipsilateral neck nodes and disseminated distant metastases
C (Dose reduction in primary tumor region and omission of contralateral elective neck nodes)	48	1	*N* = 1: oropharyngeal cancer HPV negative (tonsil)	*N* = 1 (no midline infiltration, no contralateral ND): Regional recurrence in contralateral neck node (non-irradiated neck)

### Locoregional recurrences in non-irradiated and/or dose-reduced regions

**Figure 3:**
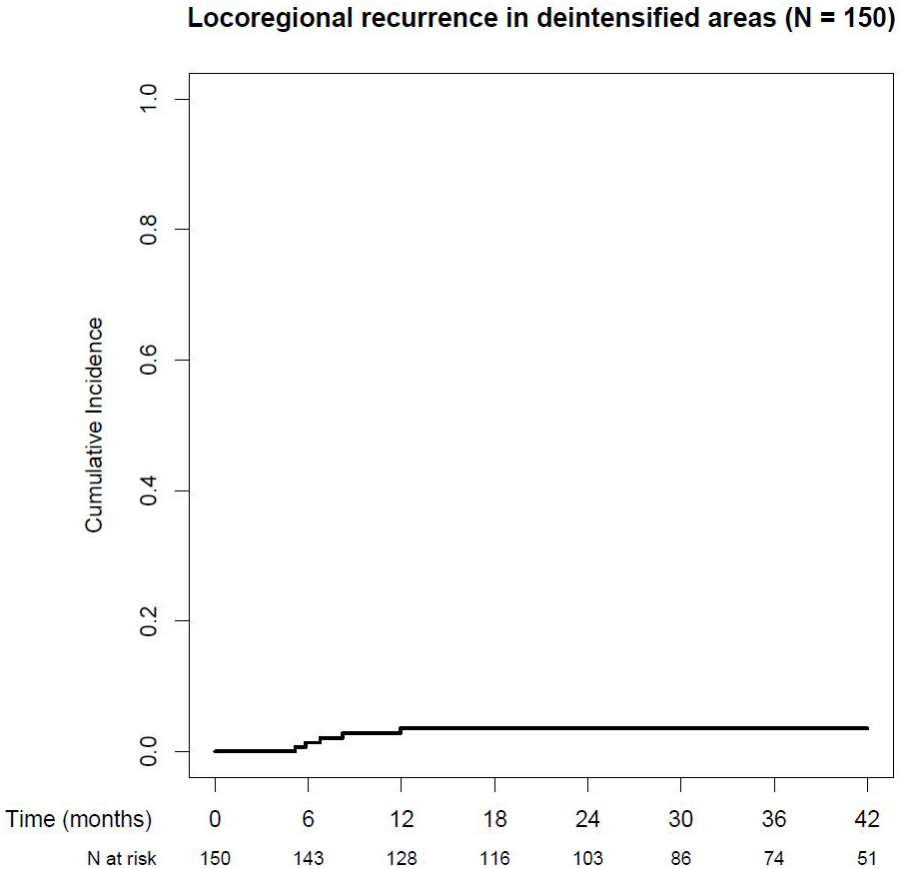
Cumulative locoregional recurrence rate in deintensified areas.

Cumulative incidence of locoregional recurrences in non-irradiated or dose-reduced regions was seen in 3.5% of all patients (95% CI: 0.4%–6.5%) after 1, 2, and 3 years (see **Figure 3**). For detailed information, see **Table 2**.

A total of 55 patients were irradiated with a reduced dose in the primary tumor region. One of these 55 patients (1.8%) developed a local recurrence in the dose-reduced region.

Only ipsilateral elective neck was irradiated in 143 patients. From these 143 patients, 4 (2.8%) patients developed a contralateral neck recurrence.

Cumulative incidence of locoregional recurrences in non-irradiated and/or dose-reduced areas was 8.2% in oral cavity cancer and 1.1% in oropharyngeal cancer after 1, 2, and 3 years.

### Prognostic factors for locoregional recurrences

**Figure 4:**
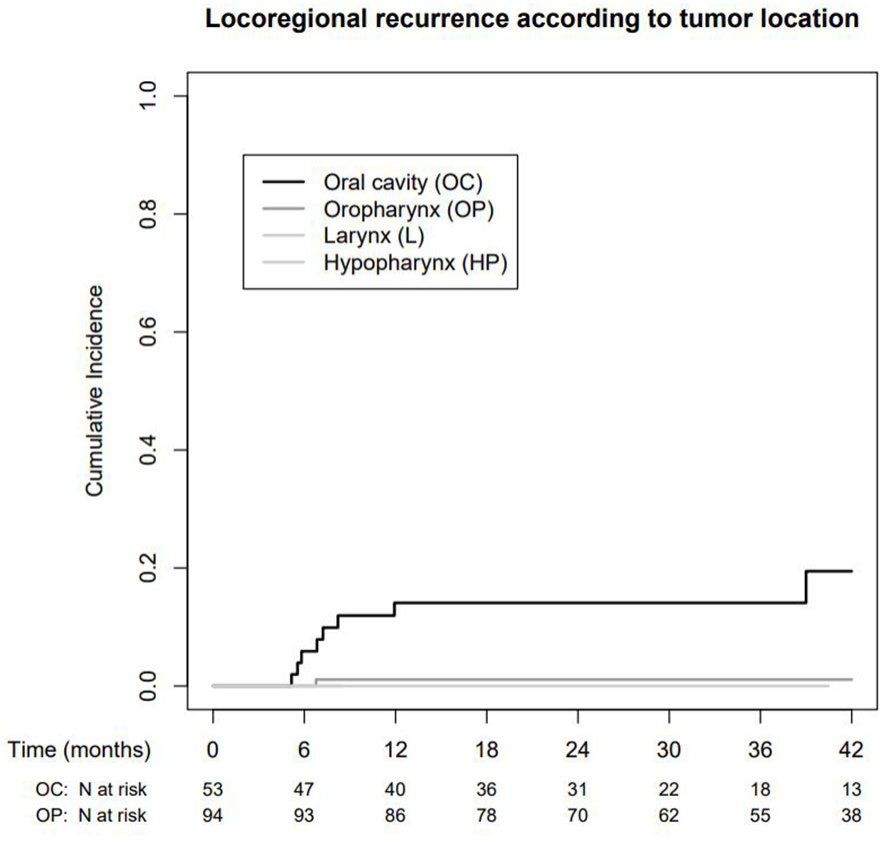
Cumulative locoregional recurrence according to tumor localisation.

In patients with oral cavity cancer, the cumulative incidence of LRR was 14.1% after 1, 2, and 3 years (95% CI: 3.8%–23.2%) and therefore significantly higher compared to patients with oropharyngeal cancer (1.1% after 1, 2, and 3 years, 95% CI: 0%–3.2%), see **Figure 4**.

Most locoregional recurrences occurred in oral cavity patients treated according to de-intensification regime B. In this patient population, univariate statistical analysis showed a trend toward a higher incidence of LRR in patients with a close (>1 mm, but <5 mm) resection margin, with perineural spread and peritumoral lymphangiosis. Midline infiltration, N status and lymph node metastases with ENE showed no correlation with LRR, see **Table 3**.

**Table 3:** Prognostic factors for locoregional recurrences in patients with oral cavity cancer treated in arm B.

Risk factor	2-year LRR	*p*-value
Lymphangiosis L0 vs. L1	10% vs. 35%	0.072
Perineural spread Pn0 vs. Pn1	10% vs. 39%	0.121
N0 vs. N1 vs. N2b	15% vs. 11% vs. 29%	0.645
Midline infiltration no vs. yes	20% vs. 8%	0.616
Extracapsular spread no vs. yes	15% vs. 20%	0.886
Close resection margin no vs. yes	6% vs. 24%	0.102

### Disease-free and overall survival

**Figure 5:**
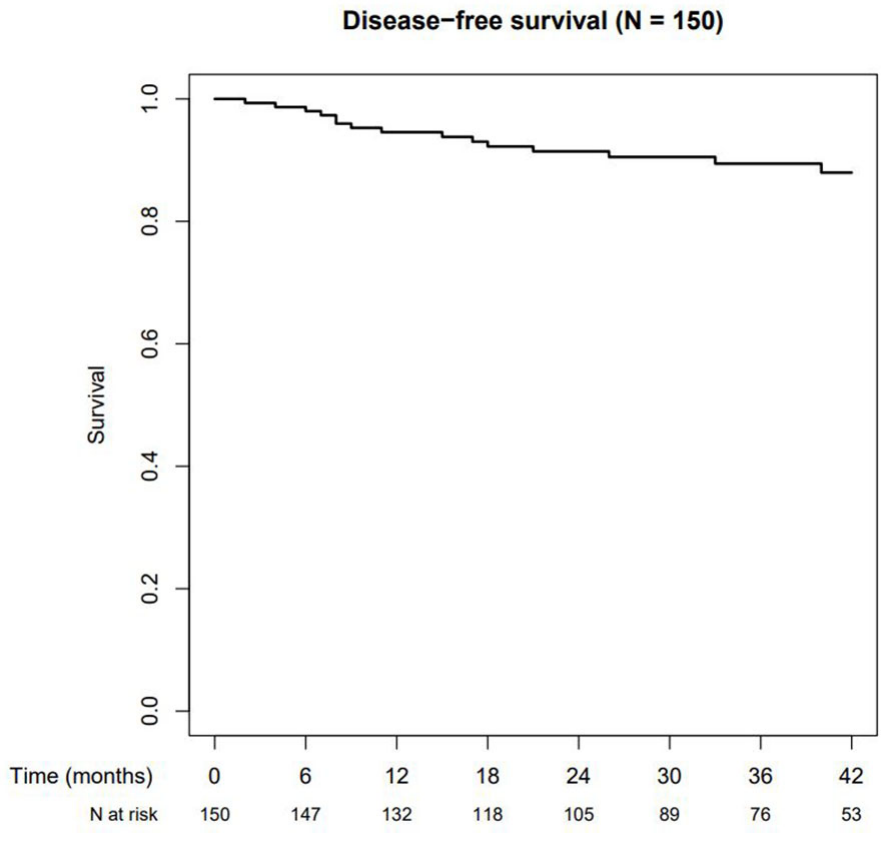
Disease-free survival.

The 1-, 2-, and 3-year disease-free survival rates (see **Figure 5**) were 95% (95% CI: 91%–98%), 92% (95% CI: 87%–96%), and 89% (95% CI: 84%–95%), and the corresponding overall survival rates were 96% (95% CI: 93%–99%), 94% (95% CI: 90%–98%), and 94% (95% CI: 90%–98%), respectively.

### Distant metastases

Cumulative incidence of distant metastases was 4.2% (95% CI: 0.8%–7.4%), 4.2% (95% CI: 0.8%–7.4%), and 5.3% (95% CI: 1.3%–9.1%) after 1, 2 and 3 years, respectively.

During the follow-up time, eight patients developed distant metastases in the following localizations: *n* = 6, lung; *n* = 1, bone; and *n* = 1, disseminated lung, pleura, and liver.

### Secondary cancer

Cumulative incidence of a second cancer was 3.6% (95% CI: 0.4%–0.67%), 6.9% (95% CI: 2.4%–11.1%), and 9% (95% CI: 3.7%–14.0%) after 1, 2, and 3 years, respectively.

A total of 17 patients developed a second cancer in the following localizations: *n* = 3, lung; *n* = 3, prostate; *n* = 2, oral cavity; *n* = 2, renal cell carcinoma; *n* = 2, contralateral tonsil; *n* = 1, colon; *n* = 1, breast cancer; *n* = 1, cholangiocarcinoma; and *n* = 1, sarcoma.

### Acute toxicity

At the beginning of radiotherapy, dysphagia according to CTC-AE v.4.0 was grade 2: 8% and grade 3: 11%, and xerostomia was grade 2: 4% and grade 3: 1%.

**Figure 6:**
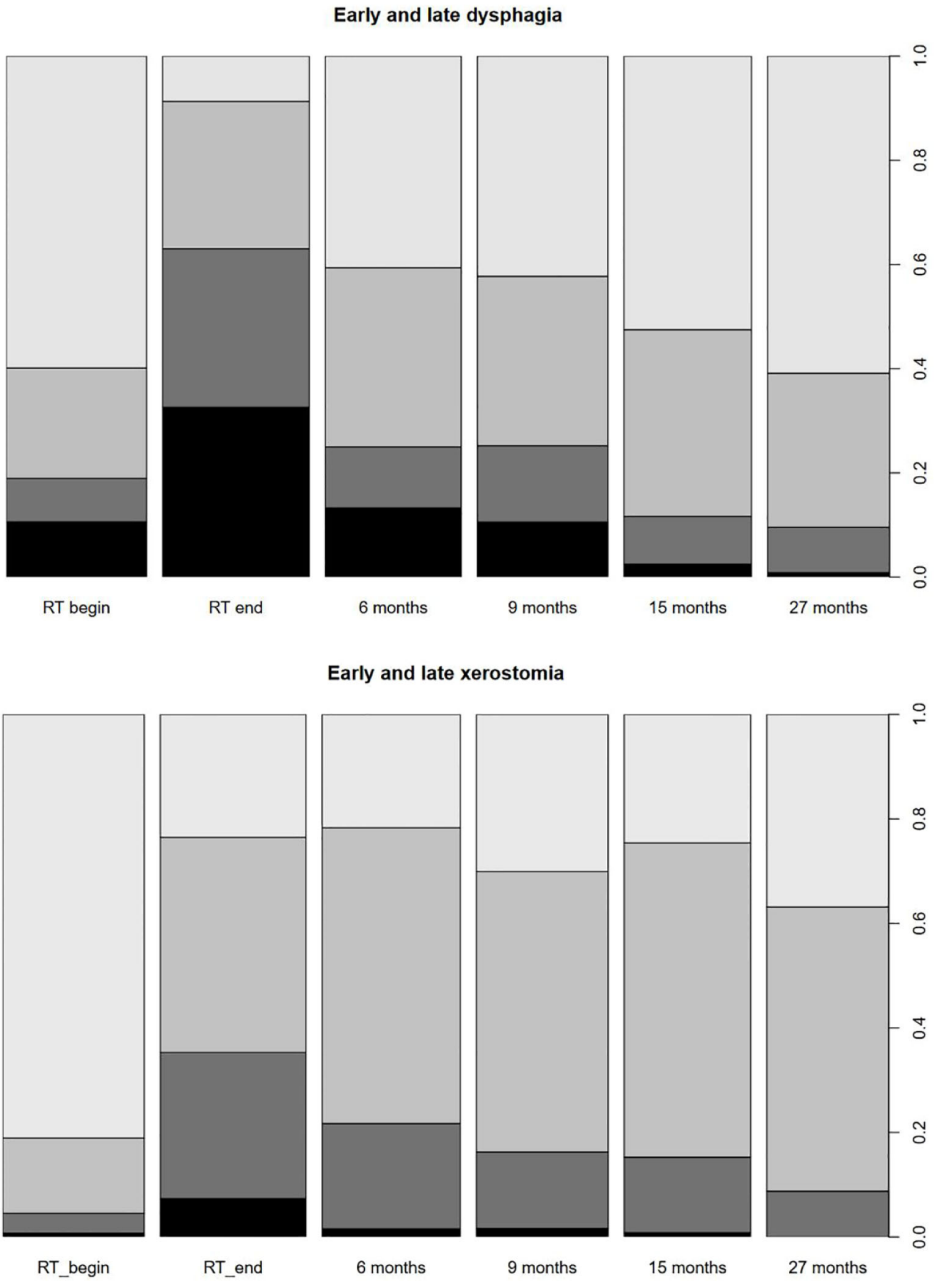
**(A)** Early and late dysphagia. **(B)** Early and late xerostomia.

In the last week of radiotherapy, acute toxicity according to CTC-AE version 4.0. was as follows: dysphagia grades 2 and 3: 33% and 30%, weight loss grades 2 and 3: 8% and 10% (see **Figures 6A, B**).

Dermatitis grades 2 and 3: 40% and 4%, xerostomia grades 2 and 3: 28% and 7%, oral mucositis grades 2 and 3: 38% and 19%, and oral pain grades 2 and 3: 29% and 7%.

No grade 4 toxicity was observed.

### Late toxicity

Grade of dysphagia and xerostomia decreased over time (see **Figures 6A, B**). After a follow-up of 9 months, grade 0, 1, 2, and 3 dysphagia was 42%, 33%, 15%, and 11% and decreased after 27 months as follows: grade 0, 1, 2, and 3 dysphagia was 61%, 30%, 9%, and 1%, respectively. After 9 months, late xerostomia was as follows: grade 0: 30%, grade 1: 54%, grade 2: 15%, and grade 3: 2%, and after 27 months, it was as follows: grade 0: 37%, grade 1: 54%, grade 2: 9%, and grade 3: 0%.

Other grade ≥3 toxicities were as follows: esophageal stenosis, 2.0%, and osteonecrosis of the mandible, 0.7%. No further grade ≥3 toxicities occurred.

Grade III° dysphagia was significantly increased in patients with concomitant chemotherapy at the end of RT [55% (with chemo) vs. 20% (without chemo)], but not late dysphagia (3% vs. 1%).

## Discussion

In this study, we investigated de-intensification of postoperative radiotherapy in 150 selected patients with HNSCC. A total of 55 patients received a dose reduction in the primary tumor region, and in 143 patients, contralateral elective neck irradiation was omitted.

Our trial showed that de-intensification of radiotherapy in a predefined patient population with head and neck cancer is possible irrespective of HPV status. Cumulative LRR after 2 years was 5.6% (95% CI 1.7%–9.2%) and therefore lower than expected. Moreover, cumulative incidence of LRR in dose-reduced or non-irradiated regions was 3.5%, whereas 2.6% of these recurrences were isolated recurrences. Therefore, the primary objective of the study was met.

Previous data showed that de-intensification of radiotherapy by dose reduction in primary tumor region or target volume reduction leads to significantly reduced dose in salivary glands and swallowing apparatus compared to standard treatment (17). Yet, it was unclear if this dose reduction is clinically relevant. Regarding late toxicity, our data show a comparably low rate of II° and III° late toxicities. Dysphagia III° decreased over time and was by 1% after a follow-up of 27 months. In comparison, in a retrospective population (6) treated with standard radiotherapy, approximately 30% of the patients suffered from grade III° late dysphagia.

An unplanned subgroup analysis showed that LRR in patients with oral cavity carcinomas who were treated with the full dose in the primary tumor region but omitting contralateral elective neck was relatively high, with most of the recurrences occurring in the primary tumor region. Locoregional recurrence in non-irradiated or dose-reduced regions was 8.2% in oral cavity cancer. There was a trend toward a higher incidence of LRR in patients with perineural spread, close resection margin, and/or peritumoral lymphangiosis. In the literature, 5-year locoregional control rates of 78% after surgery plus postoperative radiotherapy are reported (18). Other studies also show that in oral cavity cancer, LRR increases in patients with positive/close resection margins, lymphangiosis, or perineural spread (19). Therefore, it can be assumed that higher LRR in oral cavity carcinomas is because of the tumor biology. Nevertheless, de-intensified radiotherapy should be used with caution, especially dose reduction in the primary tumor region. Rather, in oral cavity cancer, consideration should be given to applying a higher dose in the primary tumor region in the presence of risk factors like close resection margin and perineral spread or lymphangiosis, even in the postoperative situation.

In tonsillar carcinoma, the NCCN guideline recommends in locally limited tumors (pT1–2pN1–N2a according to TNM classification v.7) only ipsilateral elective neck irradiation due to retrospective data (11, 13). There are only two prospective studies (8, 9) with a small sample size including 8 patients with oropharyngeal carcinoma in the definitive setting and 12 patients with oral cavity cancer in the postoperative situation investigating ipsilateral neck irradiation only (8), (9) and 37 patients with oropharyngeal cancer without midline infiltration (9). In both studies, there was no contralateral neck failure, but patients (T1–2 and N0–N2b tumor stage) were highly selected, e.g., all patients had well-lateralized tumor without midline infiltration.

Because of this paucity of prospective data, we also included patients with locally limited oropharyngeal cancer in our study. As a consequence, the retrospective data of O’Sullivan et al. (11, 13) have now been confirmed prospectively.

Moreover, data of our study show that omitting radiotherapy to the contralateral elective neck is possible not only in tonsillar carcinoma but also in other oropharyngeal carcinomas including cancer of the base of tongue and soft palate, even with midline infiltration in case of adequate selective contralateral neck dissection. In a review of Al-Mamgani et al. (12), 1,116 patients with oropharyngeal carcinomas from 11 different studies showed 2.4% of contralateral neck failure after ipsilateral radiotherapy only. However, in 9 of these 11 studies, only patients with tonsillar carcinoma were included, and in 2 of these 11 studies, only patients with tonsillar and soft palate carcinoma were included. Patients with base of tongue tumors have been excluded. In our study, we showed that 2-year locoregional control rate in patients with oropharyngeal carcinoma in all localizations and independent of HPV status is 99% (95% CI: 96.8%–100%) if strictly applying the predefined inclusion criteria.

Unfortunately, patients with laryngeal and hypopharyngeal carcinomas were underrepresented in this study and therefore our results do not allow to draw conclusions for postoperative de-intensification of radiotherapy in laryngeal or hypopharyngeal regions.

Because of the slow recruitment, the study was closed after 150 patients. The slow recruitment could have been due to the complex inclusion criteria and study design.

A shortcoming of the trial is that chemotherapy and radiotherapy were applied according to the ARO96-3 trial, which is a common therapy concept in many institutions in Germany.

The chemotherapy consists of cisplatin and 5-FU and was administered in cases of close resection margins, the presence of ECS, and with three or more affected lymph nodes. According to guidelines, mandatory indications for concurrent chemotherapy (usually with cisplatin alone) are positive resection margins and ECS. Therefore, generalizing the study data should be viewed critically.

Applied dose in the primary tumor region is 64 Gy independent of resection margin (in the current guidelines, 60 Gy in patients with complete resection and 66 Gy in patients with microscopic residual disease). In case of low risk for local recurrence, dose in the primary tumor region was reduced to 56 Gy. A dose of 56 Gy was chosen because in a previous study, we were able to demonstrate that the rate of dysphagia in HNSCC decreases the more the mean dose to the swallowing apparatus falls below 60 Gy (20). In the meantime, we know that a significantly greater dose reduction might be possible in selected patients.

Most of the published and ongoing studies investigating the de-intensification of postoperative radiotherapy are studies in patients with HPV+ oropharyngeal cancer only. ECOG-ACRIN E3311 (21), a prospective phase II study, showed that it might be possible to omit radiotherapy in low-risk patients (pT1–2pN0–N1, negative margins, according to TNM classification v.7) and to reduce radiation dose to 50 Gy in intermediate-risk patients (close margin, ECE ≤1 mm, two to four metastatic lymph nodes, perineural spread, lymph- or hemangiosis) with HPV+ oropharyngeal cancer.

In the MC1675 study (22), a prospective phase III trial, standard radiotherapy versus dose-reduced radiotherapy (30 Gy in 1.5 Gy twice daily in patients with ENE− and 36 Gy in 1.8 Gy twice daily in patients with ENE+) in combination with docetaxel on d1 and d8 was compared to standard-of-care RT (60 Gy and weekly cisplatin). The primary endpoint was toxicity (and not locoregional control) after 3 months, and the difference was not statistically significant, but regarding locoregional control in patients with more than four lymph nodes and ECE, a significantly worse locoregional control rate and reduced PFS were described.

In the AVOID Trial (23), a prospective phase II trial, omission of radiotherapy to the primary tumor region and only irradiation of involved and elective lymph node regions in tumors with pT1–2 stage and a resection margin of ≥2 mm without evidence of perineural spread were investigated. From 60 patients, 1 patient developed local recurrence in the primary tumor region, resulting in a local control rate of 98.3% after 2 years. The “incidental” median dose in the primary tumor region was 39.6 Gy and therefore much lower than the standard radiation dose.

In our trial, patients have been included independent of HPV status, but 82% of all patients with oropharyngeal cancer were HPV positive. Thus, in this patient population, according to other phase II trials (ECOG-Acrin, AVOID), radiation dose in the primary tumor region might be reduced more than we did (e.g., 50 or 40 Gy).

Contreras et al. (10) also included all kinds of HNSCC (*n* = 14: oral cavity cancer, *n* = 37: oropharyngeal cancer, *n* = 4: hypopharyngeal cancer, *n* = 16: laryngeal cancer, *n* = 1: CUP) independent of HPV status (HPV+: 49%, 43% not tested). In this prospective phase II study, omitting radiotherapy of the node negative neck was investigated. Patients with tonsillar cancer T1–2 pN0–N2a were excluded in this study. LRR in this patient population (70% mid-line infiltration) was 4%, but PFS was only 60% after 5 years. In this study, all patients had ipsilateral neck dissection and a contralateral neck dissection (92%) or a FDG-PET-CT scan (8%).

From this study and other small prospective phase II studies, we know that de-escalation of postoperative radiotherapy in selected patients is possible. However, the best strategy of de-intensification (radiation dose and/or volume reduction) remains unclear. Moreover, in all published studies, the sample size was limited and prospective phase III data with a primary endpoint regarding locoregional control rate are lacking, but may be impossible to perform as all phase II studies showed a better quality of life following de-escalation.

The fact that the cumulative incidence of second cancer after 3 years is 9% and therefore higher than the cumulative incidence of locoregional recurrence (5.6%) is in line with previous studies (6, 24).

Summarizing, we showed that target volume (omitting contralateral elective neck) and/or dose reduction in a strictly predefined patient population is safe and associated with low late toxicity rates. Therefore, de-intensification of radiotherapy should be performed if clearly defined surgical, pathological, radiological, and radiation oncological standards are fulfilled. To the best of our knowledge, this is the first study to include the biggest patient population with oropharyngeal and oral cavity cancer and investigate de-intensification of radiotherapy in the postoperative situation independent of HPV status.

As a next step, future trials may need to explore updated eligibility criteria that exclude patients who would typically be spared contralateral neck as common practice (e.g., well-lateralized tonsillar carcinoma) already, and it should be investigated if further de-escalation (e.g., only irradiation of the lymph node areas with metastatic disease and no elective neck irradiation) could be performed.

## Conclusion

The trial met its primary objective. De-intensification of radiotherapy independent of HPV status in a predefined low-risk patient population appears to result in very low rates of late toxicity without compromising locoregional control. However, in an unplanned subgroup analysis, a significantly increased risk of locoregional recurrence was observed in patients with oral cavity cancer. In these patients, de-intensified radiotherapy should be applied with caution.

## Data Availability

The raw data supporting the conclusions of this article will be made available by the authors, without undue reservation.
